# The Interaction between tRNA^Lys^_3_ and the Primer Activation Signal Deciphered by NMR Spectroscopy

**DOI:** 10.1371/journal.pone.0064700

**Published:** 2013-06-06

**Authors:** Dona Sleiman, Pierre Barraud, Franck Brachet, Carine Tisne

**Affiliations:** Laboratoire de Cristallographie et RMN biologiques, CNRS, Université Paris Descartes, Paris Sorbonne Cité, Paris, France; Institut National de la Santé et de la Recherche Médicale, France

## Abstract

The initiation of reverse transcription of the human immunodeficiency virus type 1 (HIV-1) requires the opening of the three-dimensional structure of the primer tRNA^Lys^_3_ for its annealing to the viral RNA at the primer binding site (PBS). Despite the fact that the result of this rearrangement is thermodynamically more stable, there is a high-energy barrier that requires the chaperoning activity of the viral nucleocapsid protein. In addition to the nucleotide complementarity to the PBS, several regions of tRNA^Lys^_3_ have been described as interacting with the viral genomic RNA. Among these sequences, a sequence of the viral genome called PAS for “primer activation signal” was proposed to interact with the T-arm of tRNA^Lys^_3_, this interaction stimulating the initiation of reverse transcription. In this report, we investigate the formation of this additional interaction with NMR spectroscopy, using a simple system composed of the primer tRNA^Lys^_3_, the 18 nucleotides of the PBS, the PAS (8 nucleotides) encompassed or not in a hairpin structure, and the nucleocapsid protein. Our NMR study provides molecular evidence of the existence of this interaction and highlights the role of the nucleocapsid protein in promoting this additional RNA-RNA annealing. This study presents the first direct observation at a single base-pair resolution of the PAS/anti-PAS association, which has been proposed to be involved in the chronological regulation of the reverse transcription.

## Introduction

In retroviruses, the initiation of reverse transcription is primed by a cellular tRNA that is encapsidated in viral particles. tRNA^Lys^_3_ is the natural primer of all immunodeficiency viruses, including the type 1 human immunodeficiency virus (HIV-1). Indeed, the primer tRNA is strongly bound to the genomic RNA through Watson–Crick base-pairing of its 18 3′-terminal nucleotides with the complementary viral primer binding site (PBS) (for reviews, see [1], [2]). The annealing of tRNA^Lys^_3_ to the PBS requires the action of the nucleocapsid protein (NCp7) that acts as an RNA chaperone [3], [4], [5]. HIV-1 NCp7 is a short basic protein with two zinc-finger domains that destabilizes base-pairing in the primer tRNA without opening its structure [6]. Addition of a viral template containing the PBS results in RNA-RNA complex formation and significant structural changes in both RNAs. Both basic and zinc-finger domains of NCp7 are required for proper annealing of the tRNA/RNA complex. The basic domains help to destabilize the base-pairing in the four-way junction of the tRNA structure whereas the zinc-finger domains disrupt the ternary interactions within the tRNA molecule [6], [7], [8], [9].

**Figure 1:**
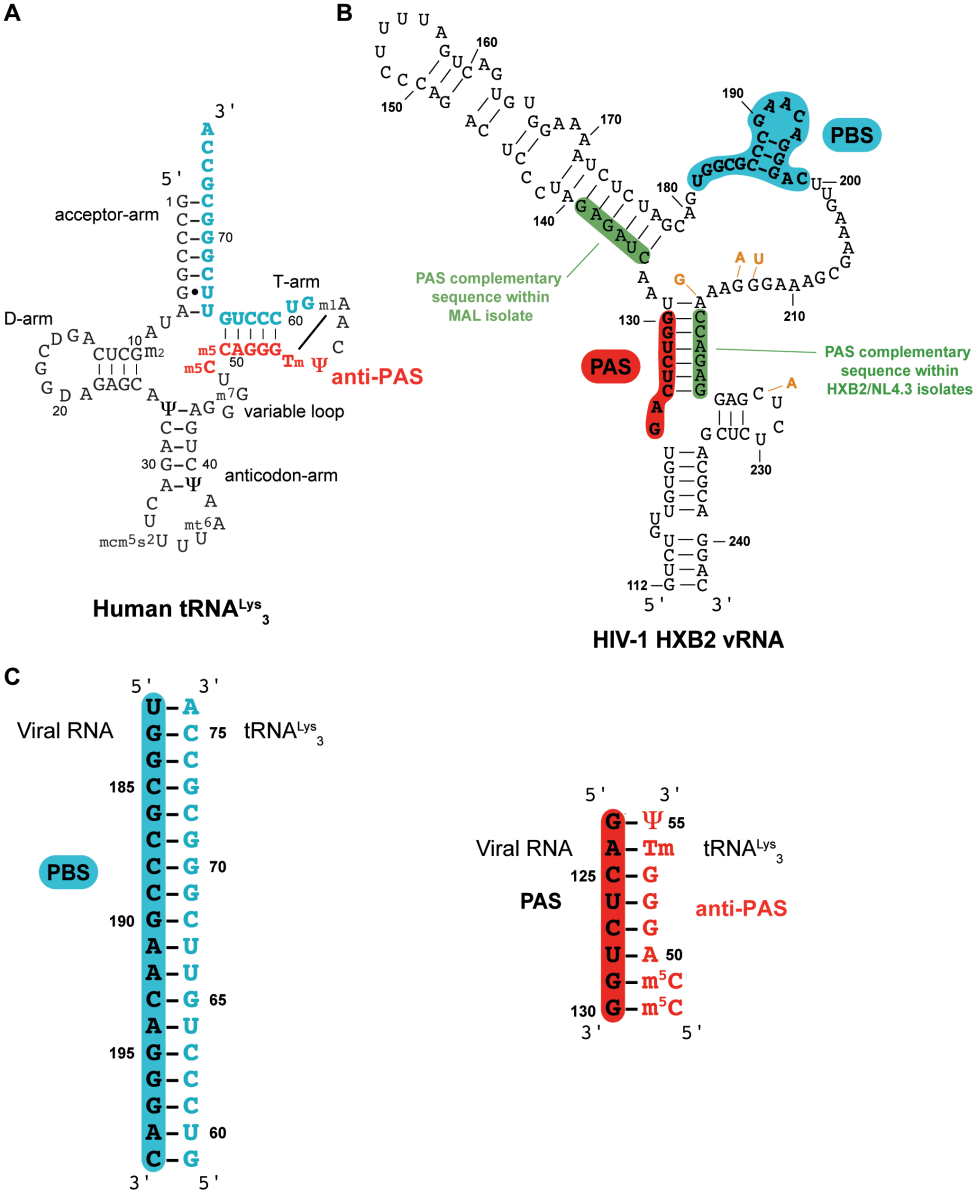
Secondary structure of A) the human tRNA^Lys^_3_, primer of the HIV-1 reverse transcription, in blue the sequence complementary to the PBS and in red the anti-PAS sequence, B) the PBS domain within the HIV-1 HXB2 isolate, in blue the PBS sequence, in red the PAS sequence and in green the sequences complementary to the PAS sequence, the nucleotides that are different between HXB2 and NL4.3 are indicated in orange, and C) the duplexes corresponding to the tRNA^Lys^_3_/PBS and to the tRNA^Lys^_3_/PAS complexes.

The specificity for tRNA^Lys^_3_ as the primer of reverse transcription is strictly maintained in HIV-1 evolution. Earlier experiments from several groups have shown that altering the PBS sequence alone is not sufficient to stably switch tRNA usage [10], [11], [12]. To improve the understanding of the exclusive usage of tRNA^Lys^_3_ as primer by HIV-1, the search for the determinants of specific tRNA^Lys^_3_ selection, other than the PBS, was the subject of extensive studies [13], [14], [15], [16], [17], [18], [19], [20], [21], [22], [23], [24], [25], [26], [27]. Additional contacts between tRNA^Lys^_3_ and the viral RNA have been naturally considered as potential secondary determinants and were proposed to play a role in reverse transcription [20], [28], [29], [30]. The different interactions (reviewed in [31]) are located in the PBS domain of the viral RNA and include an A-rich loop, a C-rich region and the primer activation signal (PAS). The A-rich loop interaction was found to play a role in the initiation of reverse transcription of the HIV-1 MAL strain but not in the NL4.3 or HXB2 isolates. By adaptation of both the PBS and PAS motifs, the HIV-1 leader could be changed to accommodate tRNA^Lys^_1,2_ as the primer of reverse transcription *in vitro* [16]. Additional studies mutating the PAS sequence in the viral RNA showed that the PAS is critically involved in stimulating tRNA^Lys^_3_-primed reverse transcription initiation in the HIV-1 HXB2 and NL4.3 strains [17], [18]. Indeed, the PAS is an 8-nucleotide sequence in the U5 region (nucleotides 123–130, Figure 1) that was proposed to be involved in a base-pairing interaction with a region located in the T-arm of tRNA^Lys^_3_ (the anti-PAS region: nucleotides 48–55, Figure 1). This interaction would thus lead to the formation of a higher order RNA structure that is suitable for stimulating initiation of reverse transcription. Indeed, the annealing of the tRNA primer to the PBS requires the opening of the acceptor and T stems of tRNA^Lys^_3_ cloverleaf structure, thus releasing the anti-PAS motif (Figure 1). However, no direct evidence of the PAS/anti-PAS association can be obtained by probing experiments [20], [32], [33]. Nevertheless, one strong argument in favour of its existence during the viral replication cycle is the very strong conservation of the PAS-like vRNA–tRNA interactions among all HIV-1 isolates, but also in SIV isolates, and even possibly in all retroviruses [16], [17], [34]. An attractive model for the regulation of HIV reverse transcription has been proposed by the group of Ben Berkhout. The PAS sequence is initially masked in the viral RNA through base-pairing in the U5 leader stem (Figure 1B). Later, this sequence anneals with the anti-PAS sequence in the primer tRNA, which stimulates the reverse transcription initiation. Altogether, the presence of the PAS enhancer motif, initially masked and repressed by base-pairing, would provide a unique mechanism for positive and negative regulation of HIV-1 reverse transcription [18]. Very recently, using FRET spectroscopy, Beerens *et al.* [35] obtained new results that are consistent with a model for tRNA annealing that involves a secondary interaction between the tRNA^Lys^_3_ molecule and the PAS sequence. In this study, the tRNA^Lys^_3_/PAS interaction appears to be dynamic and stimulated by the nucleocapsid protein [35].

In the present report, the PAS/anti-PAS interaction and the role of the nucleocapsid protein have been directly investigated *in vitro* at a single base-pair resolution using NMR spectroscopy. We show here that the PAS/anti-PAS complex exists under certain conditions that dramatically depend on the action of the nucleocapsid protein.

## Results

### RNA sequences, labelling schemes, and NMR assignments of the free RNAs

Even if important progresses have been made over the last years, NMR analysis of large RNA complexes remains technically challenging. In order to simplify the interpretation of NMR spectra, we used a selective labelling strategy in which the ^15^N labelling of tRNA^Lys^_3_ primer allowed us to only observe the NMR signals of its imino groups and not the ones of other RNA partners. In addition, the use of ^1^H-^15^N TROSY NMR experiments [36], [37] substantially improved the spectral resolution and sensitivity on these large RNA complexes. In RNA, imino protons are carried by G and U nucleotides. The intensity of imino proton NMR signals depends dramatically on their degree of protection towards exchange with the solvent. In practice, only protected imino protons that are forming stable hydrogen-bonds are visible in NMR spectra. Therefore, the NMR signal of an imino proton constitutes the signature of a base-pair within a folded RNA or between two different RNA molecules interacting to form a higher order structure. Structural information can therefore be obtained at a single base-pair resolution using NMR signals of iminos as reporter probes.

**Figure 2:**
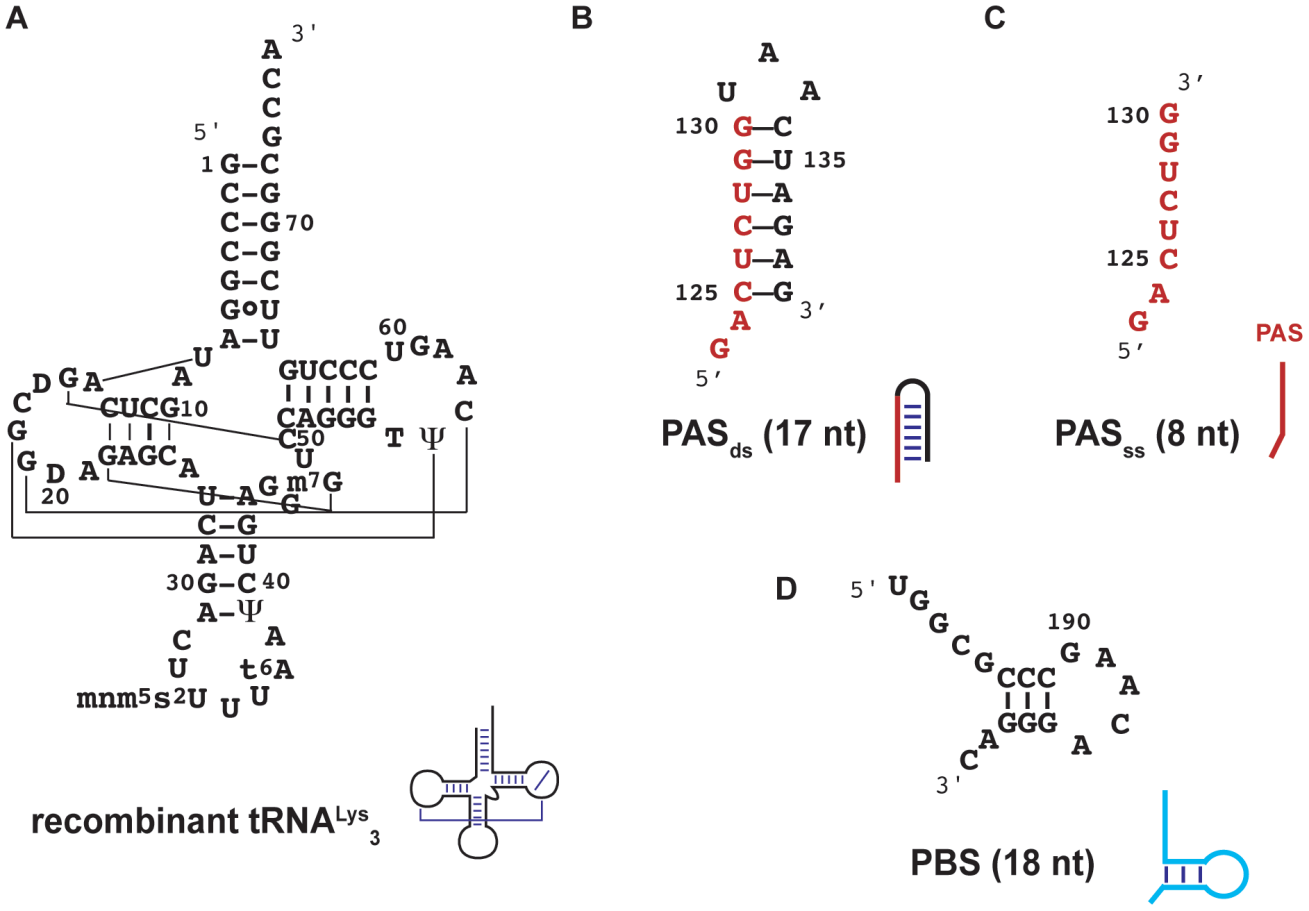
Secondary structures of RNA used in this study. A) the recombinant tRNA^Lys^_3_ expressed in *E. coli*, B) the hairpin encompassing PAS sequence, the double-stranded PAS (PAS_ds_) with the numbering of nucleotides in the HXB2 isolate, C) the single-stranded PAS sequence (PAS_ss_) and D) the PBS. A cartoon symbolizing the secondary structure of these RNAs is drawn next their names.

A similar labelling strategy using the NMR signals of the imino protons of tRNA^Lys^_3_ as reporter signals has already been successfully employed to study the secondary structure of the HIV reverse transcription initiation complex and to reveal the different steps of annealing of tRNA^Lys^_3_ to the PBS in the presence or in the absence of the nucleocapsid protein [7], [38]. In the present study, tRNA^Lys^_3_^15^N-labelling is also an appropriate tool to observe the annealing of the PAS and anti-PAS sequences (Figure 1C) as the largest number of sequential imino protons (carried by Gs and Us) belongs to tRNA^Lys^_3_, and can therefore be observed in 2D ^1^H-^15^N correlation spectra. The human tRNA^Lys^_3_ was thus produced as a recombinant tRNA in *E. coli* providing the incorporation of modified nucleotides by *E. coli* RNA modification enzymes as well as a uniform ^15^N labelling (see Materials and Methods). This recombinant tRNA (Figure 2A) bears the modified nucleotides crucial for the initiation of HIV-1 reverse transcription [21], [39]. In the case of the PAS activation, it was shown that reverse transcription primed by natural or synthetic tRNA^Lys^_3_ is similarly activated, indicating that the PAS/anti-PAS interaction is not dependent on modified nucleotides within tRNA^Lys^_3_ [18].

We performed our analysis on a model system involving critical fragments of the U5 region of HIV-1 viral RNA. The RNA molecules used to investigate the formation of the PAS/anti-PAS complex will be described below. Interestingly, the PAS sequence is involved in base-pairing within the viral RNA whatever the isolate. In the MAL isolate, the PAS sequence is part of a stem-loop structure [40] like shown in Figure 2B and S1. In the HXB2 isolate, the PAS sequence is involved in a duplex structure [41] (Figure 1B and S1). The reason for these differences comes from the fact that the PAS sequence has two potential complementary sequences within the viral RNA (see Figure 1B in green). For our NMR study, we thus chose to embed the PAS sequence in a minimal RNA hairpin (thereafter called PAS_ds_; Figure 2B) with the MAL isolate sequence and secondary structure context. This PAS_ds_ RNA hairpin was chosen as a compromise between the size of the RNA (to be kept to a minimum to facilitate NMR spectra interpretation) and the ability to mimic the natural pairing occurring in both the MAL and HXB2 isolates. We also worked with a single-stranded sequence of PAS (PAS_ss_; Figure 1B and 2C) that only contains the 8 nt of the PAS sequence. Finally, our PBS RNA sequence (PBS; Figure 1B and 2D) contained the 18 nucleotides complementary to the nucleotides at the 3′-end of tRNA^Lys^_3_. Importantly, the use of different RNA fragments to account for the different critical regions of the U5 leader RNA not only simplified the complexity of the NMR spectra for these large RNAs, but also allowed us to perform experiments with different combinations of the fragments to study the influence of the PBS sequence on the PAS/anti-PAS association (see paragraphs below).

**Figure 3:**
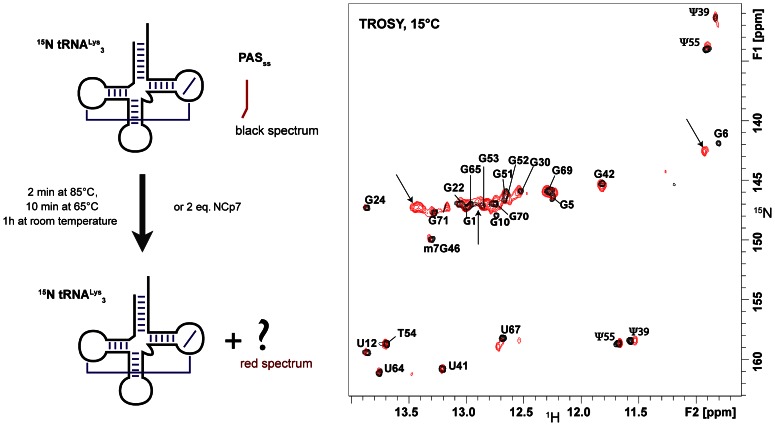
Tests of annealing of PAS_ss_ to tRNA^Lys^_3_. A) Schematic drawing of the experimental procedure, B) Superimposition of two TROSY experiments recorded at 15°C showing the imino groups of tRNA^Lys^_3_ (0.1 mM) alone (in black, reference spectrum) and after the heat-annealed procedure with 1 equivalent of PAS_ss_ (red spectrum). The arrows indicate peaks corresponding to the PAS/anti-PAS complex described in Figure 7.

**Figure 4:**
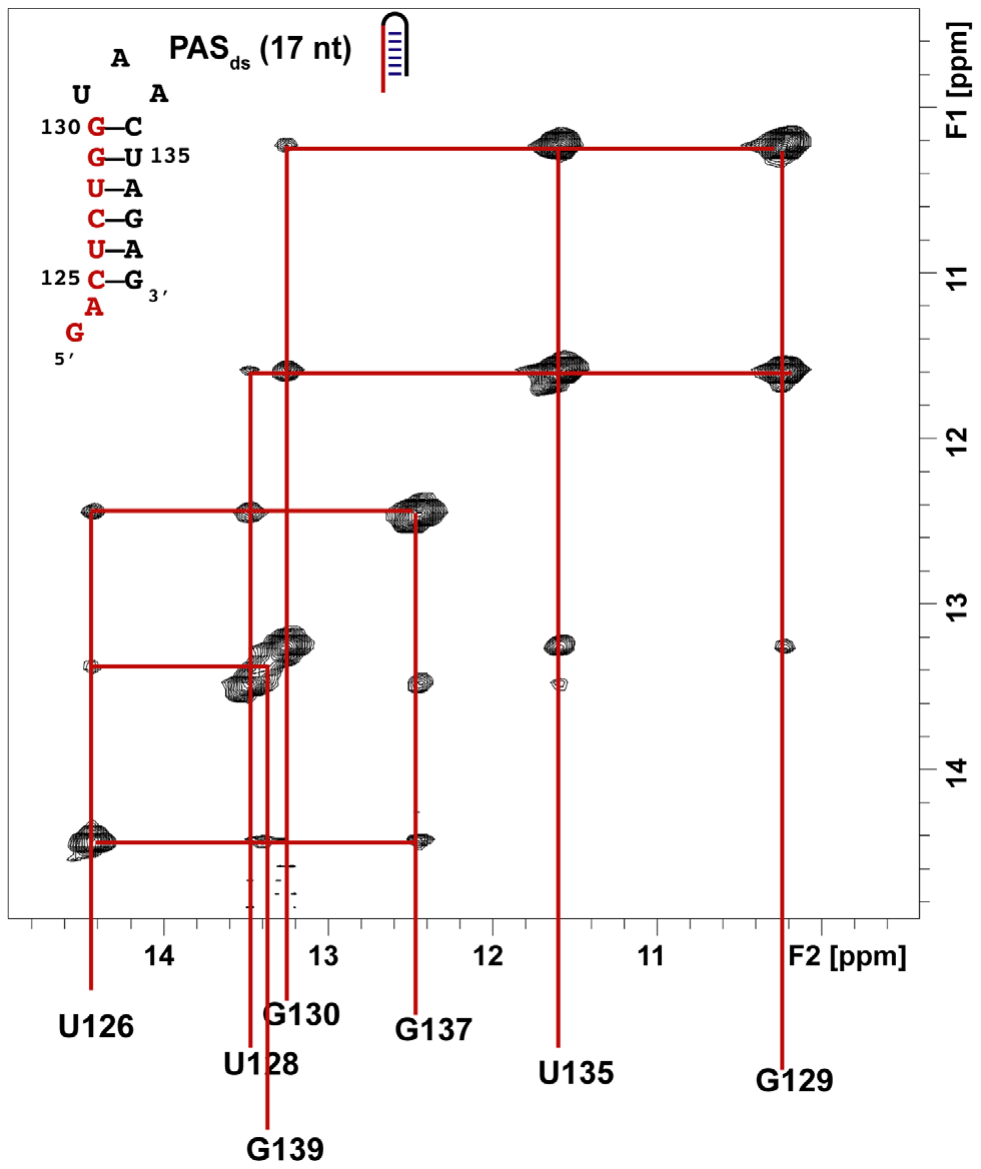
Assignment of the imino proton resonances of PAS_ds_. NOESY experiment recorded at 15°C with a mixing time of 150 ms with a sample of PAS_ds_ at 1.6 mM in a phosphate buffer (10 mM, pH 6.5) with 50 mM KCl.

Assignments of tRNA^Lys^_3_ imino groups were previously performed [6] and are indicated in Figure 3. To monitor the imino signals of PAS_ds_ in NOESY experiments, we first assigned their resonances. The PAS_ds_ hairpin contains six base-pairs and seven imino protons. Figure 4 shows the imino region of a NOESY experiment carried out at 15°C on PAS_ds_. Briefly, a GU base-pair gives rise to an intense NOE as the imino protons of the guanine and the uridine are facing each other in such a base-pairing at around 2.5 Å. In addition, in a GU pair, the imino proton of the guanine is the most shielded. Therefore, the imino proton of G_129_ was assigned to 10.21 ppm and that of U_135_ to 11.60 ppm. From this starting point, the assignment of all imino protons was straightforward and is summarized in Figure 4.

### The involvement of the PAS sequence in a doubled-stranded helix prevents it from annealing to tRNA^Lys^_3_

To investigate the annealing of the PAS signal to tRNA^Lys^_3_, we first devised two different strategies described in the materials and methods section. Briefly, the first procedure is a heat annealing-based experiment whereas the second one uses the chaperone activity of the NCp7 protein. The ^1^H-^15^N correlation NMR spectra recorded after performing these procedures on a ^15^N-labelled tRNA^Lys^_3_/PAS_ds_ mixture are identical to that of the free tRNA^Lys^_3_ (Figure S2). These experiments therefore demonstrated that tRNA^Lys^_3_ remained in the same state, *i.e.* folded on its own. Consequently, the annealing of PAS_ds_ to tRNA^Lys^_3_ could not be promoted neither by the heat-annealed nor by the NCp7-mediated procedures.

We subsequently used the single-stranded PAS sequence to conduct the two procedures in parallel. All the signals of the free tRNA^Lys^_3_ remain after these treatments (Figure 3) but the appearance of new peaks indicates that an other entity has been formed (Figure 3). We attribute the appearance of these new signals to the formation of a binary complex between tRNA^Lys^_3_ and PAS_ss_. The peaks indicated by an arrow were assigned to PAS/anti-PAS complex with the help of subsequent experiments (see paragraph below). Noticeably, the presence or the absence of NCp7 did not change the ratio between these two species (data not shown). Moreover, the comparison of the results obtained with PAS_ss_ or with PAS_ds_ strongly suggests that the embedment of the PAS sequence in base-paired region prevents it from annealing to the anti-PAS sequence of tRNA^Lys^_3_. In addition, the feature that NCp7 did not affect the ratio of the different RNA entities as compared with the heat annealing procedure is in complete accordance with NCp7 being an RNA chaperone [4], [42], [43], [44]. Taking together, these observations support the idea that the accessibility of the PAS sequence can modulate its association with the tRNA primer and thus be a regulator element of the initiation of reverse transcription [45].

### The nucleocapsid protein can promote the annealing of PAS to the anti-PAS part of tRNA^Lys^_3_ if the PBS is also annealed to tRNA^Lys^_3_

**Figure 5:**
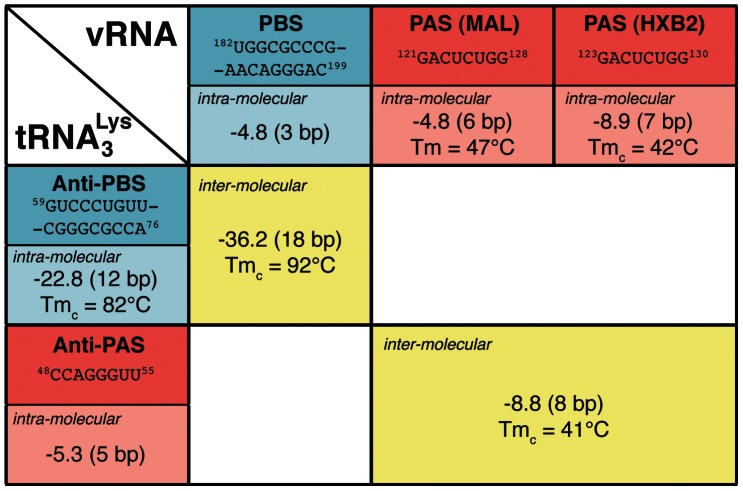
Thermodynamic stability of the interaction involving the PBS (in blue) or the PAS (in red) sequences. **Δ**G values calculated with UNAFold [57] are indicated in kcal/mol and the number of base pairs are indicated in parenthesis. The melting temperature Tm_c_ was calculated by UNAFold and the melting temperature Tm was measured by UV spectroscopy (Figure S5). See also Figure S1 for details on the different sequence and secondary structures used to model the intra-molecular interaction within the MAL and HXB2 isolates.

**Figure 6:**
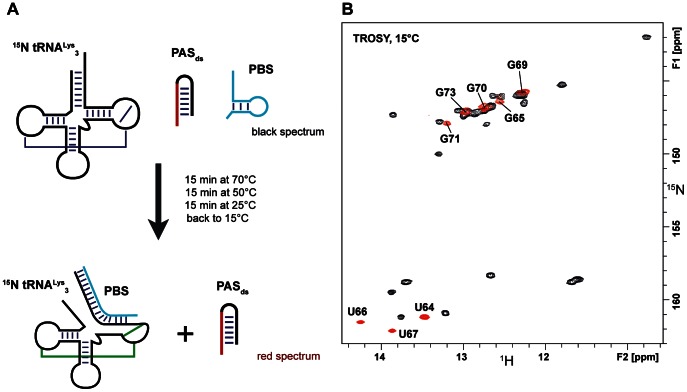
Test of annealing of PAS_ds_ to tRNA^Lys^_3_ in presence of the PBS using the heat-annealed procedure. A) Schematic drawing of the annealing procedure, B) Superimposition of two TROSY experiments recorded at 15°C showing the imino groups of tRNA^Lys^_3_ alone (in black, reference spectrum) and after the heat-annealed procedure with 1 equivalent of PBS and PAS_ds_ (red spectrum).

From a thermodynamic point of view, the annealing of tRNA^Lys^_3_ to the PBS is strongly favoured (Figure 5, **Δ**G  = −36.2 kcal/mol and Tm_c_  = 92°C). In addition, the annealing of tRNA^Lys^_3_ to the PBS will partially open the tRNA structure and thus increase the accessibility to the anti-PAS sequence. Therefore, in order to investigate whether the PBS could influence the annealing of the PAS sequence with tRNA^Lys^_3_, we decided to conduct the heat-annealed procedure in the presence of the PBS. Figure 6 shows the NMR footprint experiment resulting from this procedure. The assignment of tRNA^Lys^_3_/PBS imino groups was previously published [7]. The imino groups of tRNA^Lys^_3_ (black spectrum – Figure 6) have disappeared to give rise to imino groups of tRNA^Lys^_3_ involved in base-pairing with the PBS (red spectrum – Figure 6). A NOESY experiment (data not shown) substantiates that the PAS_ds_ is still free as its imino groups are still observable at the chemical shifts of the imino groups of the free PAS_ds_ like in Figure 4. In conclusion, when we mixed ^15^N-tRNA^Lys^_3_ to the PBS and to the PAS_ds_ at the same time, the heat-annealed complex is composed of the PBS bound to tRNA^Lys^_3_ and the PAS remains free in solution (Figure 6). This complex is stable and does not change with time.

**Figure 7:**
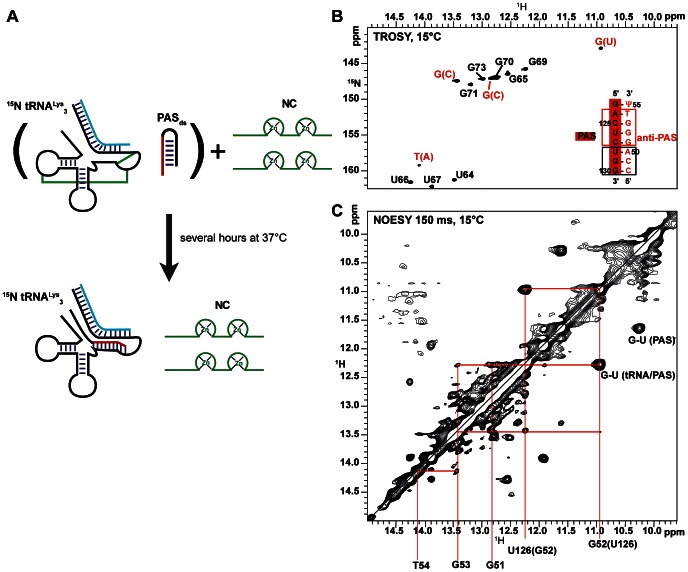
Assignment of the NCp7-mediated tRNA^Lys^_3_/PBS/PAS_ds_ complex (0.5 **mM of each component).** A) Schematic drawing of the experimental procedure, B) TROSY experiment recorded at 15°C showing the imino groups of tRNA^Lys^_3_ (0.5 mM) after the NCp7-mediated procedure described in A), C) NOESY experiment recorded on the same sample as in A at 15°C with a mixing time of 150 ms showing the region of the imino groups. The red square indicates base-pairings assigned thanks to the use of the TROSY (A) and the NOESY (B) experiments whereas the black square indicates base-pairings assigned from the comparison of NOESY experiments.

Subsequently, we added two equivalents of NCp7 to this mixture and left it for several hours at 37°C. Interestingly, new signals in addition to those originating from the tRNA^Lys^_3_/PBS complex further appeared in the NMR footprint experiment (Figure 7) indicating the formation of new base-pairs. Because the PBS and the PAS sequences are not labelled, only the imino groups of tRNA^Lys^_3_ are observable in ^1^H-^15^N TROSY experiments whereas all the imino signals from tRNA^Lys^_3_, PBS and PAS are observable in ^1^H-^1^H NOESY experiments. Moreover, N1 of Gs and N3 of Us and Ts can be easily distinguished by their ^15^N chemical shift. Therefore, we could easily assign the four new signals to three ^15^N-labelled guanines and one ^15^N-labelled pyrimidine involved in new base-pairings and belonging to tRNA^Lys^_3_. A NOESY experiment enabled us to assign the new signals to the imino groups of the PAS/anti-PAS complex as highlighted on Figure 7C. In short, the G_52_-U_126_ pair is readily assigned since the ^15^N-labelled G at 10.93 ppm in the proton dimension is connected through an intense NOE with a non-labelled U at 12.27 ppm as expected for a G-U pair. Both nucleotides are connected with a ^15^N-labelled guanine at 13.44 ppm that is further connected to the T resonating at 14.10 ppm. In addition, the ^15^N chemical shift of T_54_ at 159.16 ppm and the sequence of the PAS/anti-PAS complex further supports that this peak corresponds to the T_54_ of tRNA^Lys^_3_ involved in the complex. The last signal corresponding to a guanine in the TROSY spectrum (12.85 ppm-147.04 ppm) was assigned to G51 since its imino proton gives an NOE to both imino protons of the G_52_-U_126_ pair. The imino groups carried by the PAS sequence (U128, G129, G130) were more difficult to assign since they are not labelled and therefore do not give rise to peaks in the TROSY experiment. We proceeded using a stepwise strategy: first we identified in the NOESY experiment of the mixture (Figure 7) the cross-peaks originated from the PAS_ds_ alone and that from the tRNA^Lys^_3_/PBS complex using previously recorded NOESY experiments (Figure S3). Then, the comparison of these three NOESY experiments revealed imino cross-peaks originating from the tRNA/PAS complex (black peaks alone). In short, we assigned U128 to 13.63 ppm, G129 to 13.26 ppm and G130 to 12.37 ppm by superimposing and comparing these three NOESY experiments (Figure S3). Only the G-Ψ base-pair, located at one extremity of the PAS/anti-PAS interaction region, was not observed in our experiments. These data clearly demonstrate for the first time the existence of a PAS/anti-PAS association at a single base-pair resolution.

It is interesting to notice that in addition to the signals of the PAS annealed to tRNA^Lys^_3_, the signals of the free PAS_ds_ are still observable in the NOESY experiment, and this even though tRNA^Lys^_3_ and PAS have been mixed at a 1∶1 ratio. It is easily observable on the GU pairs. The free and the bound PAS contain each a GU pair that gives rise to an intense peak appearing at a different chemical shift for the free and the bound PAS (Figure 7C). These characteristic GU peaks represent therefore a clear signature that not all the PAS_ds_ has annealed to tRNA^Lys^_3_. The comparison of the intensity of these two GU cross-peaks in the NOESY experiment indicates that approximately 50% of the PAS_ds_ is annealed to tRNA^Lys^_3_. Whatever the salt concentration, the amount of NCp7 (not more than 4 equivalents to avoid aggregation), we did not succeed in obtaining more than 50% of PAS/anti-PAS complex. We could not rule out that the amount of NCp7 added in our samples is not sufficient to reach higher amount of the PAS/anti-PAS complex. Briefly, we want here to mention that no exchange cross-peaks between individual imino protons in the free and annealed form of the PAS RNA are observed in the different NOESY spectra (Figure 7 and S3). This absence is most probably attributable to a rapid exchange of the unprotected imino protons with the solvent during the inter-conversion between the free and bound forms of the PAS. In conclusion, the PAS_ds_ signal can anneal to the anti-PAS segment of tRNA^Lys^_3_ when the PBS is present under the action of the nucleocapsid protein. In contrast with the annealing of the PBS to tRNA^Lys^_3_, only a fraction of the PAS (∼50%) is annealed to tRNA^Lys^_3_.

For the single-stranded PAS sequence, we cannot quantify the amount of PAS_ss_/anti-PAS complex that is formed, since the free single-stranded PAS_ss_ has no observable imino proton. However, we investigated the possibility to make this complex with the heat-annealing or the NCp7-mediated procedures. Interestingly, the heat-annealed complex is present in sufficient amount to give observable NMR signals when 1 equivalent of PAS_ss_ is mixed with equimolar quantity of tRNA^Lys^_3_ and PBS (Figure S4). The addition of PAS_ss_ at 5 equivalents did not change the resulting NMR footprint. The addition of two equivalents of NCp7 protein led to the same results suggesting that NCp7 would be crucial for destabilizing the base-pairings within PAS_ds_, but would be dispensable in the case of an unpaired PAS sequence, as seen here with the PAS_ss_ RNA. Indeed, the destabilization of RNA helices is a well-known feature of the NCp7 chaperone activity [46], [47], [48], [49]. For this reason, we ultimately investigated the interaction between NCp7 and PAS_ds_ by 1D ^1^H NMR. Interestingly, we observed an important broadening of all imino proton NMR signals indicating that NCp7 binds non-specifically to PAS_ds_ and is likely to induce a destabilization of the hairpin (Figure S6).

## Discussion

In this report, we provide direct evidence at a molecular level and with a single base pair resolution of base-pairings between the PAS in the viral RNA and the anti-PAS in tRNA^Lys^_3_. In addition, we showed that NCp7 promotes the annealing of the PAS sequence to its complementary anti-PAS sequence within the primer tRNA^Lys^_3_. Importantly, we were unable to simulate the action of the nucleocapsid protein with a simple heat-annealing procedure when the PAS sequence is engaged in a double-stranded region, accentuating the importance of NCp7 in this RNA structural rearrangement. Our results are in agreement with a recent study [35] showing a two-fold increase of annealed tRNA^Lys^_3_ molecules to the template viral RNA containing the PAS sequence in the presence of NCp7 compared to a heat-annealing procedure. Moreover, differences between viral RNA/tRNA^Lys^_3_ complex promoted by heat or by NCp7 have been noticed, in particular, the conformational heterogeneity of this complex becomes smaller in the presence of NCp7 [35]. This study thus suggests that NCp7 facilitates tRNA annealing to the PAS motif. Our work provides further and complementary evidences at the level of single base-pairs, regarding the formation of the PAS/tRNA^Lys^_3_ complex using small fragments of viral RNA.

### Thermodynamic analysis

Thermodynamic considerations are important to properly describe and understand these large structural rearrangements involving three different RNA molecules. The formation of the complex between tRNA^Lys^_3_ and the PBS is largely thermodynamically favoured (Figure 5, **Δ**G = −36.2 kcal/mol, Tm_c_ = 92°C). As a result, the tRNA/PBS complex can easily be promoted by both the heat-annealed and the NCp7 procedures [7].

The situation concerning the formation of the binary complex between tRNA^Lys^_3_ and the PAS is very different. tRNA^Lys^_3_ on its own is more stable than paired with the PAS sequence. Indeed, the free energy (**Δ**G) reaches −22 kcal/mol with a melting temperature Tm_c_ calculated by UNAFold above 80°C whereas for the PAS/anti-PAS complex, **Δ**G is only −8.8 kcal/mol with a Tm_c_ around 40°C (Figure 5). In addition, the free PAS is already involved within the viral RNA in quite stable secondary structures (**Δ**G = −4.8 kcal/mol, Tm = 47°C for the MAL isolate and **Δ**G = −8.9 kcal/mol, Tm_c_ = 42°C for the HXB2 strain; Figure 5). As a result, the tRNA/PAS binary complex is clearly disfavoured and we showed that it cannot be promoted neither by the heat-annealed nor the NCp7-mediated procedures (Figure S2). The situation is different in the case of the binary complex between tRNA^Lys^_3_ and the single-stranded PAS molecule (PAS_ss_). Indeed, in this case, the thermodynamic parameters of the tRNA/PAS complex have not changed, but the PAS sequence cannot fold on its own and only 6 bp (Figure 5 and 1A) at the level of the tRNA^Lys^_3_ T-arm need to be disrupted. However, the T-arm is stacked on the acceptor arm forming a long helix (12 bp) that further stabilizes this region. This could explain why the single-stranded PAS can only be partially annealed to the primer tRNA^Lys^_3_ (Figure 3).

Finally, the situation governing the formation of the ternary complex between tRNA^Lys^_3,_ the PBS, and the PAS is of interest. The tRNA^Lys^_3_/PBS complex is thermodynamically greatly favoured thereby freeing the tRNA^Lys^_3_ anti-PAS sequence. The PAS sequence within the HXB2 viral RNA or annealed with the anti-PAS region of tRNA^Lys^_3_ are of similar thermodynamic stability. Altogether, these thermodynamic considerations can help explaining why the association of the PAS sequence with the anti-PAS region of tRNA^Lys^_3_ can only be observed in the presence of the PBS (Figure 6), unless one uses the single-stranded PAS molecule (Figure S4). Indeed, as NCp7 acts as an RNA chaperone, it helps remodelling RNA structures towards the most stable assembly. Here, the strand exchange property of NCp7 is definitely crucial to promote the formation of the ternary complex (tRNA^Lys^_3_/PBS/PAS) that was shown to stimulates the initiation of reverse transcription [17], [18]. For the PAS/anti-PAS interaction, the fact that the intra-molecular PAS association within the viral RNA and the inter-molecular association with tRNA^Lys^_3_ are of comparable stability (see Figure 5), can very likely explain why we did observed a 50/50 mixture of the PAS sequence annealed with tRNA^Lys^_3_ or with “itself” in the PAS_ds_ hairpin.

### Relevance for HIV-1 initiation of reverse transcription

Obviously, the use of small viral RNA fragments and the absence of reverse transcriptase cannot reproduce all the events occurring during the initiation of HIV-1 reverse transcription. But, replacing our new findings in the context of previous works showing that the PAS sequence stimulates the initiation of HIV-1 reverse transcription [17], [18] is, however, very informative. First, we confirmed that the PAS/anti-PAS interaction requires the annealing of tRNA^Lys^_3_ to the PBS thus, these new base-pairings will not drive the annealing of tRNA^Lys^_3_ to the PBS. Consequently, our results are in agreement with works that demonstrated that the PAS sequence had no effect on the hybridization of the tRNA primer on the PBS [18]. Secondly, the interaction between tRNA^Lys^_3_ and the PAS motif is dynamic [35], and probably short-lived during the reverse transcription, since the reverse transcriptase must penetrate the PAS/anti-PAS helix during the early elongation phase [50]. Our observation that only a fraction of the PAS sequence is annealed to tRNA^Lys^_3_ is definitely related to its low relative stability, its dynamic behaviour and might certainly be put in relation with its transient nature *in vivo*. It also probably explains why chemical probing did not succeed to uncover this interaction [32], [33]. Interestingly, in the case of the A-rich loop of the viral RNA of MAL isolates that interacts with the anticodon loop of tRNA^Lys^_3_, NMR did not succeed in observing it [7], [38] whereas a number of chemical probing studies did [20], [21], [22], [51]. Thirdly, the PAS sequence in the HIV-1 genome is occluded by base-pairing in the U5 leader stem, and thus, needs to be free for the interaction with the tRNA primer. We showed that the nucleocapsid, as expected, plays a major role to promote its annealing to the anti-PAS sequence, most probably by destabilizing the PAS duplex and promoting strand exchange between the viral RNA and tRNA^Lys^_3_.

Finally, the presence of the PAS motif that is temporarily repressed by base-pairing provides a unique mechanism for regulation of HIV-1 reverse transcription. It was speculated that this mechanism may preclude premature reverse transcription in virus-producing cells such that the vRNA genome is copied only after it is appropriately packaged into virions [18], [52], [53]. Because NCp7 is released from the Gag precursor protein during maturation of virion particles, this mechanism will ensure the precise timing for activation of reverse transcription. tRNA packaging and annealing to the PBS in viral particles is not dependent on Gag processing [54], whereas efficient tRNA primer extension is. PAS accessibility may therefore regulate reverse transcription in the viral life cycle.

## Conclusions

We showed in this report that base-pairings between the PAS in the viral RNA and the anti-PAS in tRNA^Lys^_3_ can be observed by NMR under certain conditions. In our hands, the PAS/anti-PAS interaction is positively influenced by several factors: (i) the annealing of tRNA^Lys^_3_ to the PBS, which results in the partial opening of the tRNA structure and therefore increases the accessibility to the anti-PAS sequence; (ii) the presence of the nucleocapsid chaperone protein, which might destabilize the initial PAS base-pairing within the viral RNA and favour RNA strand exchange. On the other hand, it is disfavoured by the molecular inaccessibility of the PAS sequence as a result of its embedment in a base-paired region within the viral RNA. Altogether, the PAS/anti-PAS interaction appears to be a new illustration of the chaperone activity of the NCp7 protein. In addition, our data highlight the power of NMR experiments to map global RNA folds and demonstrate that NMR is a powerful tool to characterize annealing processes in detail.

## Materials and Methods

### Sample Preparation

^15^N-labelled tRNA^Lys^_3_ was expressed in *E. coli* (JM101TR strain) from a recombinant plasmid and purified as previously described [39]. Small fragments of HIV viral RNA namely the PBS (18 nucleotides), PAS_ds_ (17 nucleotides) and PAS_ss_ (8 nucleotides) were purchased from Dharmacon research with 2′-O-bis (acetoxyethoxy)-methyl (ACE) protection. The samples were deprotected following manufacturer recommendations and lyophylised. They were then dissolved in a phosphate buffer (10 mM, pH 6.5) with 50 mM KCl. The PBS and the PAS_ds_ hairpin were folded by heating at 90°C for 5 min and snap-cooling on ice. The sequences of RNA samples used in this study are indicated in Figure 2. The sequence of the PAS_ds_ hairpin corresponds to the sequence and secondary structure context of the MAL isolate, and was chosen as a compromise between the size of the RNA (to be kept to a minimum for the NMR study) and the ability to mimic the natural pairing occurring in both the MAL and HXB2 isolates (See also Figure S1).

The recombinant NCp7 protein (HIV-1 strain NL4.3, 55 residues) was overexpressed from the bacterial expression vector pRD2, which contains the NCp7 coding region from HIV-1 strain NL4-3 subcloned into pET-3a (Novagen, WI). This plasmid was kindly provided by M.F. Summers [55], [56]. pRD2 was transformed into *E. coli* strain BL21(DE3)pLysE and the overexpressed protein was purified as previously described [55], [56].

### Heat-annealed and NCp7-mediated procedures

In the standard heat-annealed procedure, RNA samples were heated at 85°C for 2 minutes, next incubated at 65°C for 10 minutes before a slow cooling to room temperature and putting them in the NMR tube. In the experiment of Figure 6, as we acquired NMR spectra at each temperature, the sample containing tRNA^Lys^_3_ (0.44 mM), PAS_ds_ and PBS at ratio 1∶1∶1 was first heated at 70°C in the NMR spectrometer for the time necessary to acquire 1D ^1^H NMR spectrum (roughly 15 minutes in total). Then the temperature of the sample was progressively decreased to 50°C, 40°C, 25°C and 15°C in the NMR spectrometer and a 1D ^1^H NMR spectrum was acquired at each temperature. At 15°C, a NOESY and a TROSY NMR experiments were recorded to analyse the RNA complex in the tube.

In the NCp7-mediated procedure, two equivalents of NCp7 were added to RNA samples and the mixture was then heated at 37°C for 30 min to several hours. Both procedures were performed in the NMR buffer (10 mM KPO4 pH 6.5) containing 50 mM KCl or 125 mM KCl. The same results were obtained at both salt concentration.

Thermodynamic stabilities and melting temperatures reported in Figure 5 were calculated using the UNAFold web server (http://mfold.rna.albany.edu/) [57]. The melting temperature of PAS_ds_ was measured by UV spectroscopy measuring the absorbance at 260 nm from 15°C to 80°C (Figure S5).

### NMR spectroscopy

NMR data were measured on a Bruker Avance DRX600 spectrometer equipped with a TCI cryoprobe. 1D ^1^H NMR spectra were recorded using a watergate sequence for solvent suppression [58]. For samples at a concentration of 0.1 mM, 512 scans were usually acquired. ^1^H-^15^N TROSY experiments [36], [37] were carried out with 128 t1 increments and 2048 t2 data points per increment. The t1 dimension was acquired with the echo-antiecho method. The spectral widths were 3600 Hz and 1900 Hz in the proton and the nitrogen dimensions, respectively. NOESY experiments with a mixing time of 150 ms were recorded at 15°C using a watergate sequence for solvent suppression [58]. 256 t1 increments and 2048 t2 data points per increment were recorded. The t1 dimension was acquired with the States-TPPI method. The spectral widths were 12600 Hz in both dimension. Spectra were processed using TOPSPIN (Bruker) and analysed with TOPSPIN or CcpNMR (http://www.ccpn.ac.uk/).

The interaction between ^15^N-tRNA^Lys^_3_ and others partners were carried out at a concentration of 0.45 mM when we needed to acquire a NOESY experiment and at a concentration of 0.1 mM when only a TROSY experiment was necessary to analyze the RNA complex in the NMR sample.

## Supporting Information

Figure S1 **RNA sequences used to model the secondary structure context of the PAS sequence within A) the MAL isolate and B) the HXB2 isolate.** These RNA fragments were used to derive the thermodynamic parameters for the PAS sequence within the two different viral RNA isolates (See Figure 5).
(TIF)

Figure S2 **Tests of annealing of PAS_ds_ to tRNA^Lys^_3_.** A) Schematic drawing of the experiment, B) Superimposition of two TROSY experiments recorded at 15°C showing the imino groups of tRNA^Lys^_3_ (0.1 mM) alone (in black, reference spectrum) and after the heat-annealed procedure with 1 equivalent of PAS_ds_ (red spectrum).
(TIF)

Figure S3 **Superimposition of three NOESY experiments recorded at 15°C with a mixing time of 150** **ms showing the imino-imino region, in blue for PAS_ds_, in red for a mixture of tRNA^Lys^_3_, PAS_ds_ and PBS for which the annealing between the tRNA^Lys^_3_/PBS was promoted, in black for a mixture of tRNA^Lys^_3_, PAS_ds_, PBS, NCp7 for which the annealing tRNA^Lys^_3_/PBS/PAS_ds_ was promoted (same experiment as** **Figure** **7** **).** The NOESY cross-peaks for GU base-pairs within PAS_ds_, and tRNA^Lys^_3_/PAS complex are indicated. The assigment of the imino groups within the PAS/anti-PAS complex is indicated in purple for ^15^N-signals and the assignment for black cross-peaks corresponding to non-labelled imino groups are in orange.
(TIF)

Figure S4 **Annealing of PAS_ss_ to tRNA^Lys^_3_ in the presence of PBS.** Superimposition of two TROSY experiments recorded at 15°C showing the imino groups of tRNA^Lys^_3_ (0.1 mM), in black: in complex with PBS (1 equivalent) and PAS_ss_ (1 equivalent) after the heat-annealed procedure; and in red: in complex with PBS (1 equivalent) and PAS_ss_ (1 equivalent) after the NCp7-mediated procedure. Only the GC regions are shown since the T54 imino group was not observed in these experiments.
(TIF)

Figure S5 **UV melting curve of the PAS_ds_.**
(TIF)

Figure S6 **Region of the imino protons of the ^1^H NMR spectra of the PAS_ds_ alone (0.16** **mM) and of the PAS_ds_ (0.16** **mM) mixed with NCp7 at 1∶1 ratio.** The spectra were recorded at 15°C using a watergate sequence to [58] to achieve water signal suppression.
(TIF)
