# Supplementary figures and images for: The Interaction between tRNALys 3 and the Primer Activation Signal Deciphered by NMR Spectroscopy
Source: PLoS One. 2013 Jun 6;8(6):e64700. doi: 10.1371/journal.pone.0064700 (PMC3675109; doi:10.1371/journal.pone.0064700)

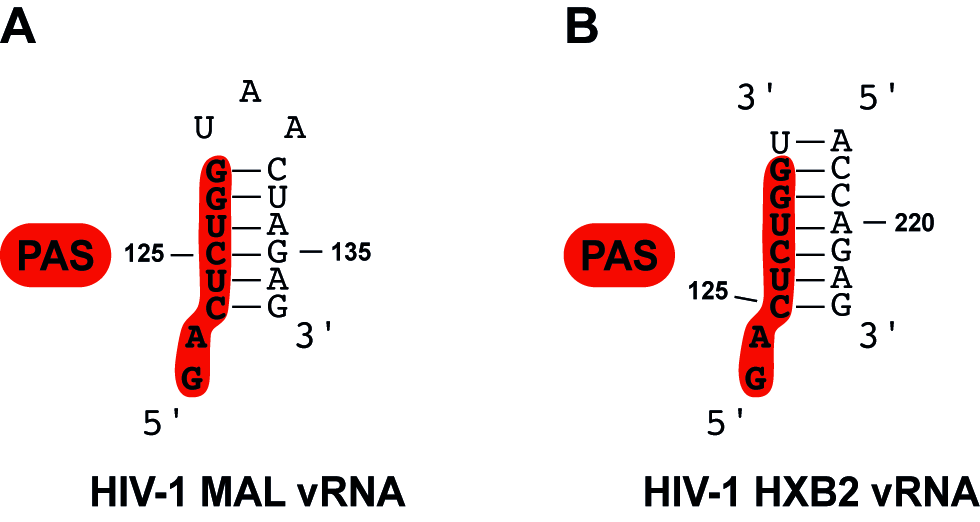

Supplement: Figure S1 — RNA sequences used to model the secondary structure context of the PAS sequence within A) the MAL isolate and B) the HXB2 isolate. These RNA fragments were used to derive the thermodynamic parameters for the PAS sequence within the two different viral RNA isolates (See Figure 5). (TIF) [file pone.0064700.s001.tif]

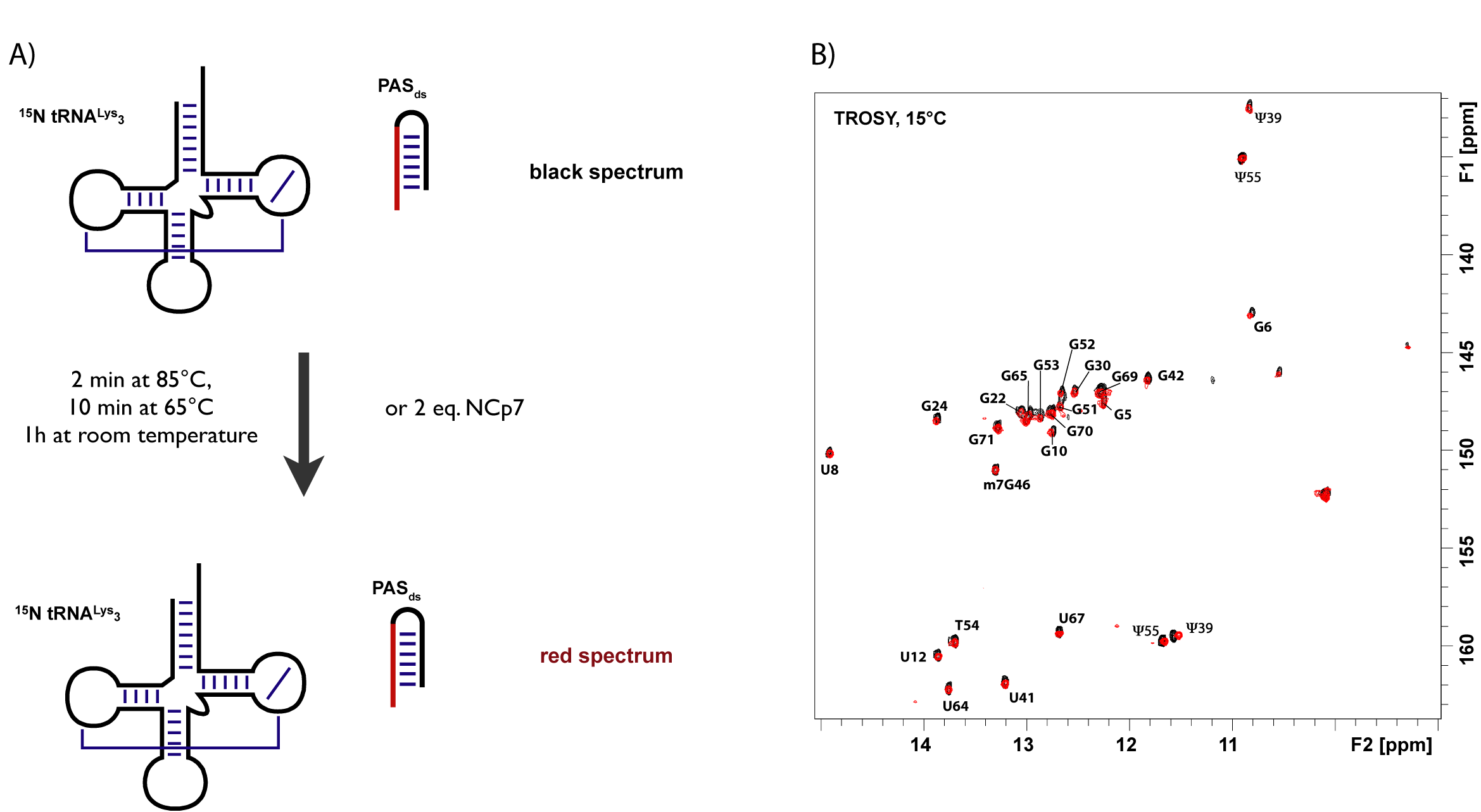

Supplement: Figure S2 — Tests of annealing of PASds to tRNALys3. A) Schematic drawing of the experiment, B) Superimposition of two TROSY experiments recorded at 15°C showing the imino groups of tRNALys 3 (0.1 mM) alone (in black, reference spectrum) and after the heat-annealed procedure with 1 equivalent of PASds (red spectrum). (TIF) [file pone.0064700.s002.tif]

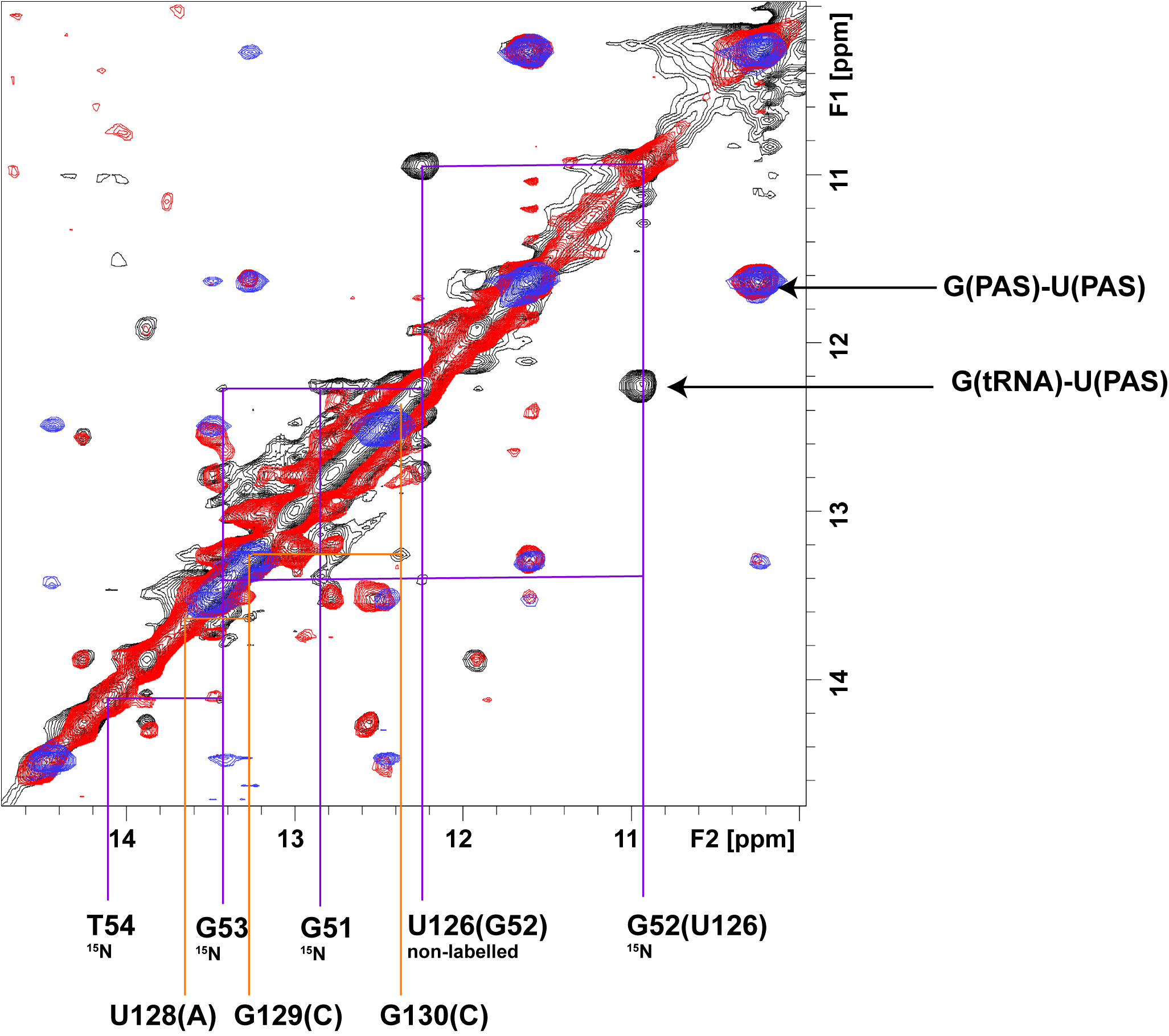

Supplement: Figure S3 — Superimposition of three NOESY experiments recorded at 15°C with a mixing time of 150 ms showing the imino-imino region, in blue for PASds, in red for a mixture of tRNALys3, PASds and PBS for which the annealing between the tRNALys3/PBS was promoted, in black for a mixture of tRNALys3, PASds, PBS, NCp7 for which the annealing tRNALys3/PBS/PASds was promoted (same experiment as Figure 7 ). The NOESY cross-peaks for GU base-pairs within PASds, and tRNALys 3/PAS complex are indicated. The assigment of the imino groups within the PAS/anti-PAS complex is indicated in purple for 15N-signals and the assignment for black cross-peaks corresponding to non-labelled imino groups are in orange. (TIF) [file pone.0064700.s003.tif]

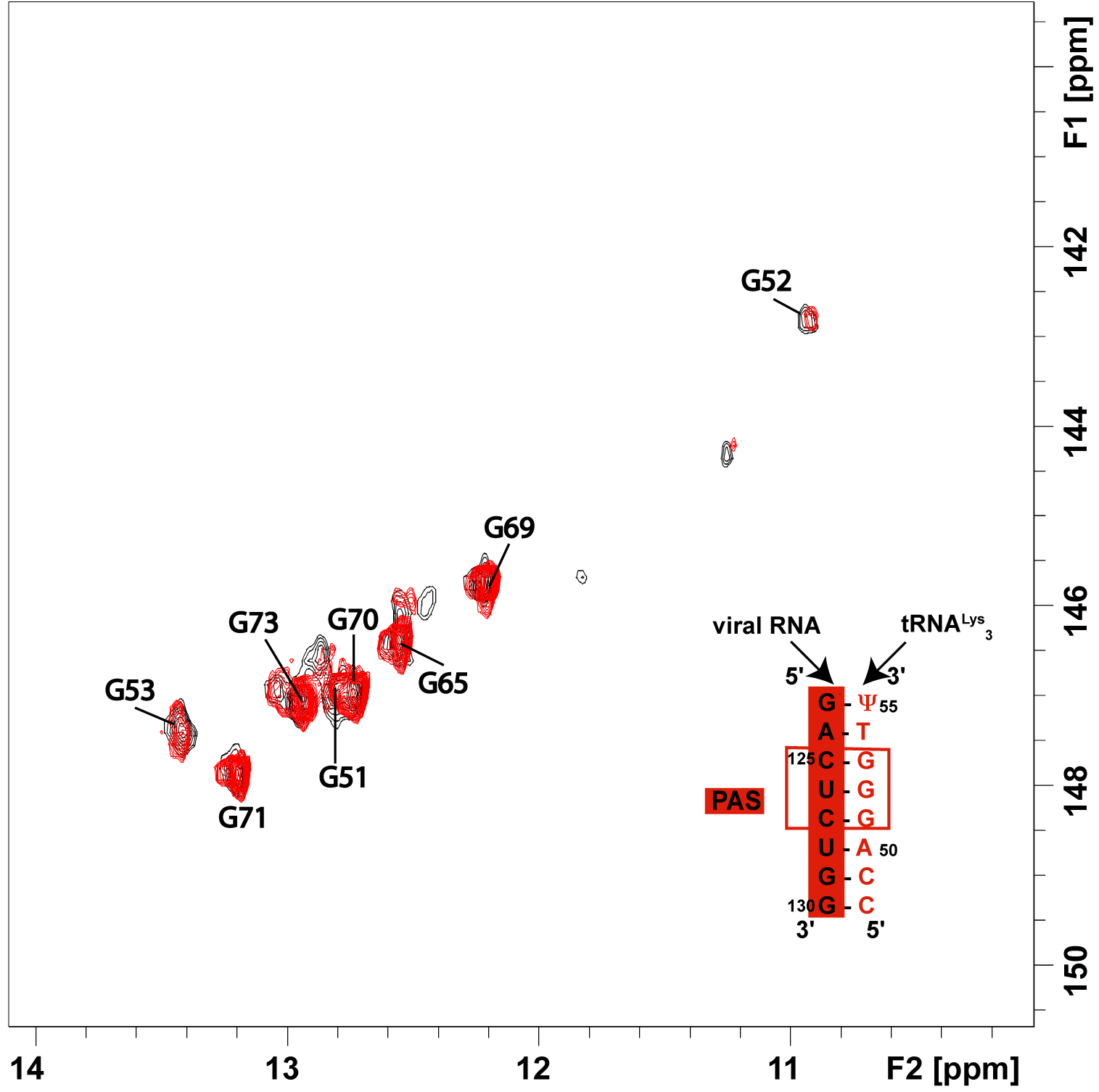

Supplement: Figure S4 — Annealing of PASss to tRNALys3 in the presence of PBS. Superimposition of two TROSY experiments recorded at 15°C showing the imino groups of tRNALys 3 (0.1 mM), in black: in complex with PBS (1 equivalent) and PASss (1 equivalent) after the heat-annealed procedure; and in red: in complex with PBS (1 equivalent) and PASss (1 equivalent) after the NCp7-mediated procedure. Only the GC regions are shown since the T54 imino group was not observed in these experiments. (TIF) [file pone.0064700.s004.tif]

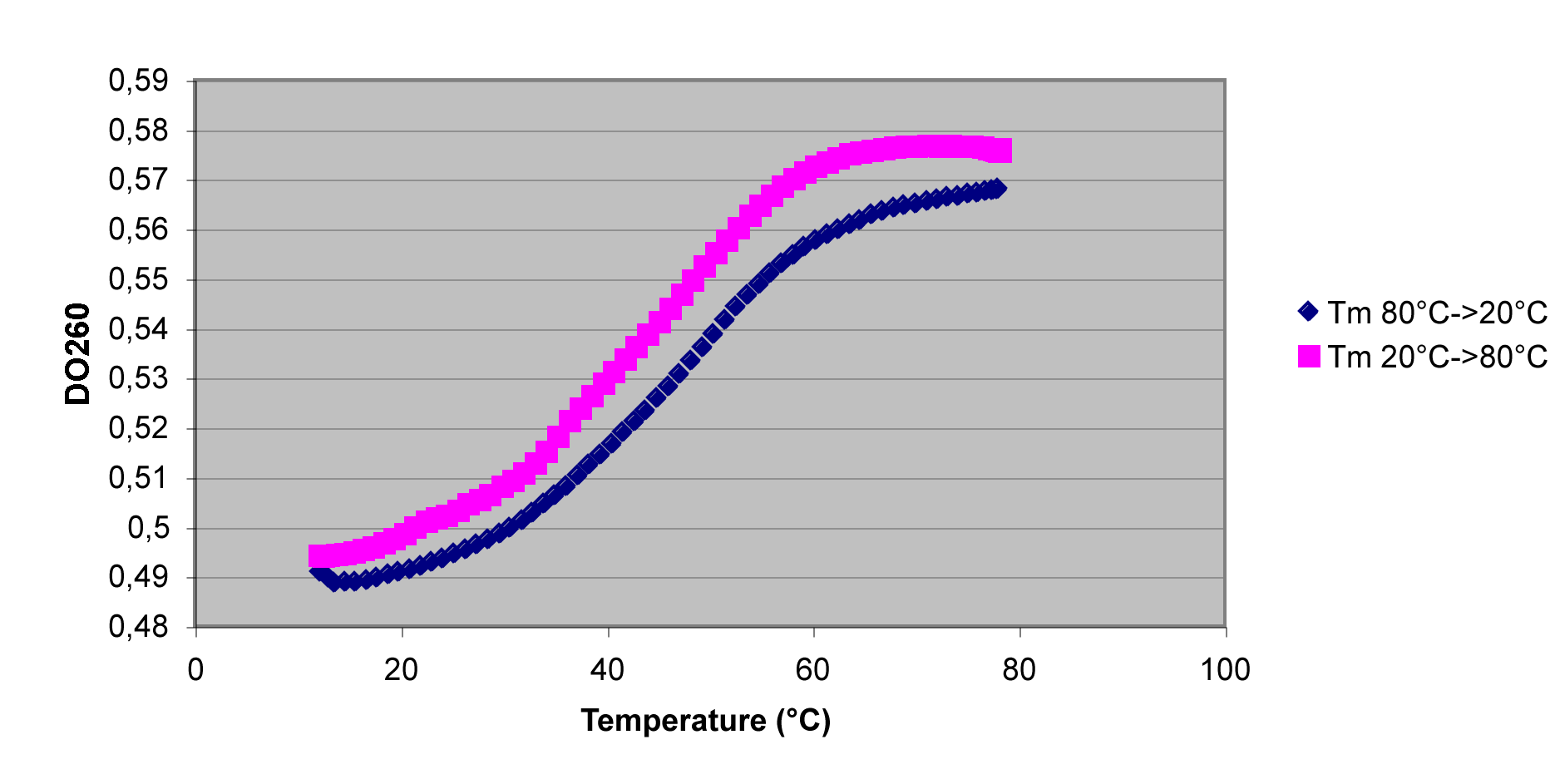

Supplement: Figure S5 — UV melting curve of the PASds. (TIF) [file pone.0064700.s005.tif]

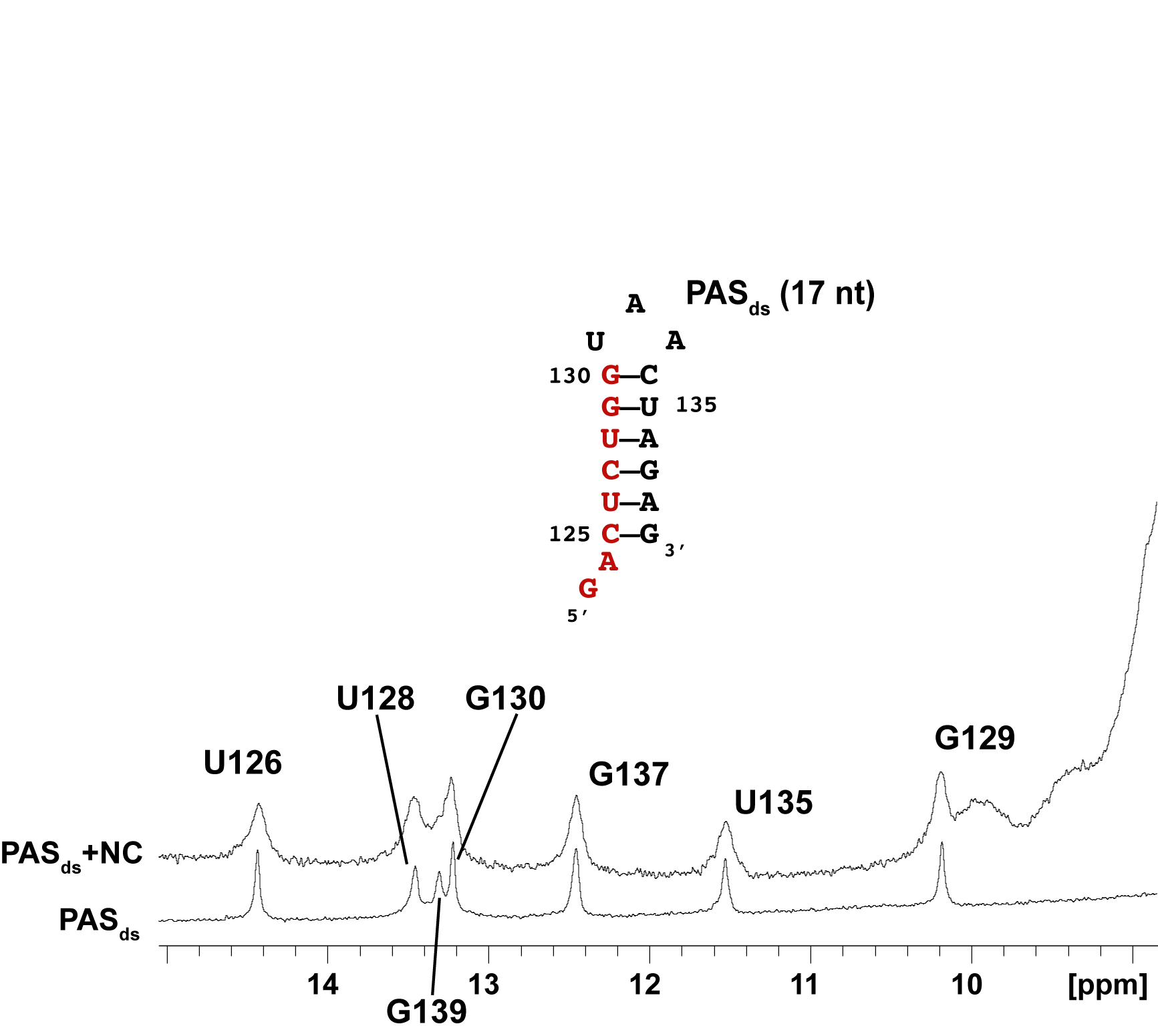

Supplement: Figure S6 — Region of the imino protons of the 1H NMR spectra of the PASds alone (0.16 mM) and of the PASds (0.16 mM) mixed with NCp7 at 1∶1 ratio. The spectra were recorded at 15°C using a watergate sequence to [58] to achieve water signal suppression. (TIF) [file pone.0064700.s006.tif]
